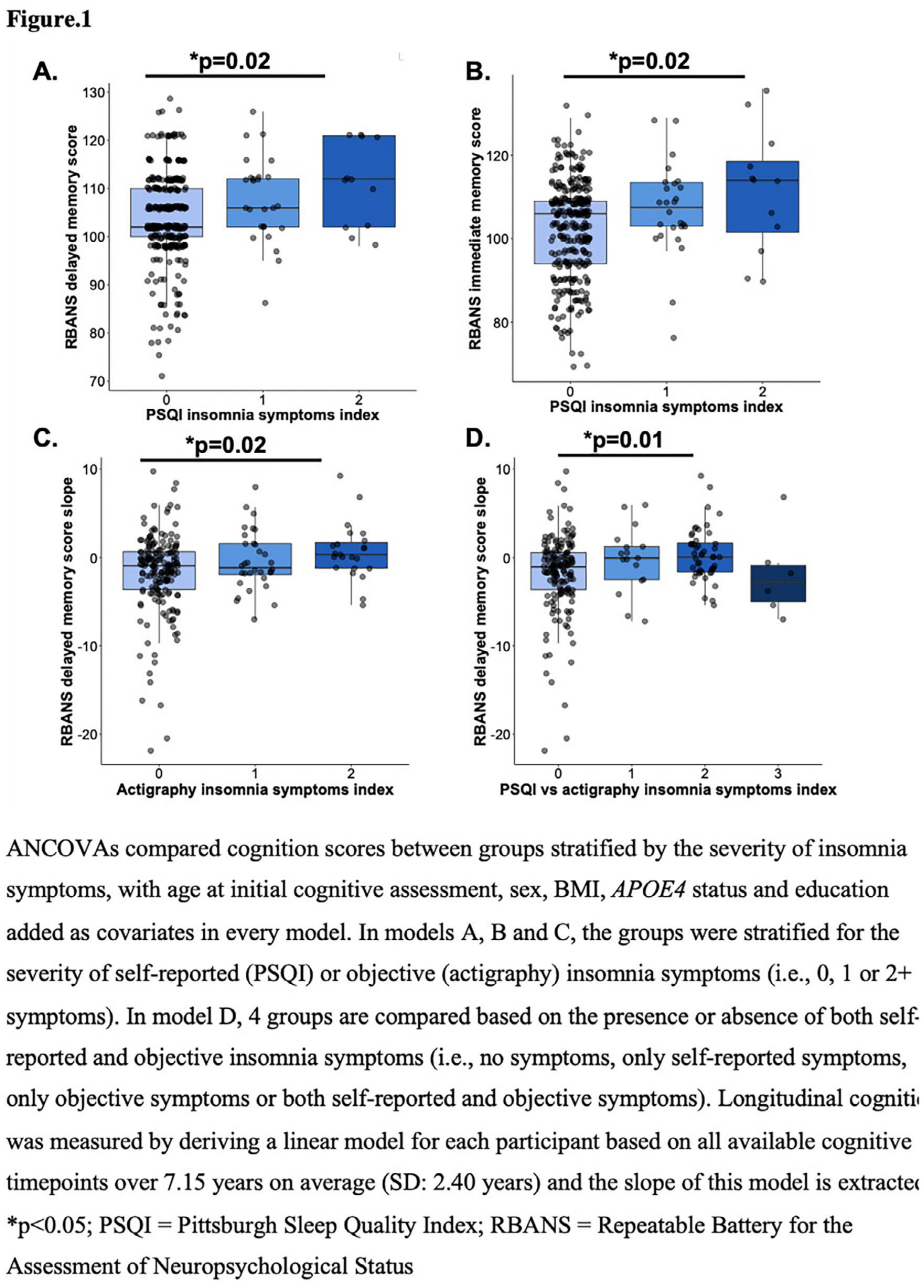# Self‐reported and objectively assessed insomnia symptom severity and cognitive markers in dementia‐free older adults at‐risk for Alzheimer's disease

**DOI:** 10.1002/alz70856_103128

**Published:** 2025-12-26

**Authors:** Bery Mohammediyan, Sylvia Villeneuve, Béatriz Oliveira, John C.S. Breitner, Judes Poirier, Erlan Sanchez, Claire André, Géraldine Rauchs, Andree‐Ann Baril, PREVENT‐AD Research Group

**Affiliations:** ^1^ Center for advanced research in sleep medicine, Research Center of the CIUSSS‐NIM, Montreal, QC, Canada; ^2^ Department of Medicine, Université de Montréal, Montreal, QC, Canada; ^3^ Douglas Mental Health University Institute, Montreal, QC, Canada; ^4^ Department of Psychiatry, McGill University, Montreal, QC, Canada; ^5^ McGill Centre for Integrative Neuroscience, McGill University, Montreal, QC, Canada; ^6^ Université de Montréal, Montréal, QC, Canada; ^7^ Center for Advanced Research in Sleep Medicine, Hôpital du Sacré‐Coeur de Montréal, CIUSSS‐NIM, Montréal, QC, Canada; ^8^ Douglas Mental Health University Institute, Centre for Studies on the Prevention of Alzheimer's Disease (StoP‐AD), Montréal, QC, Canada; ^9^ Department of Psychiatry, McGill University, Montréal, QC, Canada; ^10^ Centre Intégré de Traumatologie, Hôpital du Sacré‐Coeur de Montréal, CIUSSS‐NIM, Montreal, QC, Canada; ^11^ Clinical Research Center, Lyon Institute For Aging, Charpennes Hospital, Hospices Civils de Lyon, Villeurbanne, France; ^12^ Normandie Univ, UNICAEN, INSERM, U1237, PhIND “Physiopathology and Imaging of Neurological Disorders”, NeuroPresage team, Cyceron, Caen, France; ^13^ Inserm, Inserm UMR‐S U1237, Université de Caen‐Normandie, GIP Cyceron, Boulevard H. Becquerel, Caen, France; ^14^ Research Center of the CIUSSS‐NIM, Hôpital du Sacré‐Coeur de Montréal, Montreal, QC, Canada; ^15^ Department of Medicine, Université de Montréal, Montréal, QC, Canada

## Abstract

**Background:**

We investigated whether insomnia may be associated with changes in cognition over time among older adults at elevated risk of Alzheimer's disease (AD).

**Methods:**

We studied 348 dementia‐free older adults (mean age: 65.74 ± 5.57; 72% female) from the PREVENT‐AD cohort, all at elevated AD risk due to family history of AD. At baseline, a self‐reported insomnia symptoms index was computed based on the Pittsburgh Sleep Quality Index (PSQI). The presence of insomnia symptoms was defined as sleep onset latency >30min ≥3 times/week OR sleep maintenance is difficult ≥3 times/week AND these sleep disturbances interfere with daily life. A subsample of 240 participants also underwent objective sleep assessments (7‐day actigraphy). Difficulty with sleep maintenance on actigraphy was defined as ≥3 times/week with a wake after sleep onset >60min or a sleep efficiency <78% (1.5 SD of sample). Cognition was assessed both at baseline and yearly for a total follow‐up of 7.15 years on average (SD: 2.40 years), and included measures of immediate and delayed memory, attention, and total score on the Repeatable Battery for Assessment of Cognitive Status (RBANS). First, baseline and longitudinal cognition scores were compared between 3 groups stratified for self‐reported insomnia symptoms severity (i.e., 0, ≤1 and 2+ symptoms) using ANCOVAs. The same analyses were performed between 3 groups stratified for objectively defined insomnia symptoms. Finally, cognition scores were compared between 4 groups stratified by the severity of both self‐reported and objective insomnia symptoms (i.e., no symptoms, only self‐reported symptoms, only objective symptoms or both self‐reported and objective symptoms).

**Results:**

Individuals with ≥2 self‐reported insomnia symptoms had higher baseline immediate and delayed memory scores than individuals with ≤1 insomnia symptoms (*p* = 0.02, Figure 1A; *p* = 0.02, Figure 1B). Actigraphy‐indicating ≥2 insomnia symptoms had higher longitudinal delayed memory scores than individuals with ≤1 insomnia symptom (*p* = 0.02, Figure 1C). Individuals whose insomnia symptoms were observed only with actigraphy had the greatest longitudinal delayed memory scores (*p* = 0.01, Figure 1D).

**Conclusion:**

Counterintuitively, in individuals at elevated risk of AD, more self‐reported and objective insomnia symptoms were associated with better cognitive scores. This may reflect protective lifestyle factors that improve cognition but also disrupt sleep patterns and sleep satisfaction.